# Blast Shock Wave Mitigation Using the Hydraulic Energy Redirection and Release Technology

**DOI:** 10.1371/journal.pone.0039353

**Published:** 2012-06-20

**Authors:** Yun Chen, Wei Huang, Shlomi Constantini

**Affiliations:** 1 BrightstarTech, Inc., Clarksburg, Maryland, United States of America; 2 Uniformed Services University of the Health Sciences, Bethesda, Maryland, United States of America; 3 Department of Pediatric Neurosurgery, Dana Children's Hospital, Tel-Aviv Medical Center, Tel Aviv University, Tel Aviv, Israel; University of South Florida, United States of America

## Abstract

A hydraulic energy redirection and release technology has been developed for mitigating the effects of blast shock waves on protected objects. The technology employs a liquid-filled plastic tubing as a blast overpressure transformer to transfer kinetic energy of blast shock waves into hydraulic energy in the plastic tubings. The hydraulic energy is redirected through the plastic tubings to the openings at the lower ends, and then is quickly released with the liquid flowing out through the openings. The samples of the specifically designed body armor in which the liquid-filled plastic tubings were installed vertically as the outer layer of the body armor were tested. The blast test results demonstrated that blast overpressure behind the body armor samples was remarkably reduced by 97% in 0.2 msec after the liquid flowed out of its appropriate volume through the openings. The results also suggested that a volumetric liquid surge might be created when kinetic energy of blast shock wave was transferred into hydraulic energy to cause a rapid physical movement or displacement of the liquid. The volumetric liquid surge has a strong destructive power, and can cause a noncontact, remote injury in humans (such as blast-induced traumatic brain injury and post-traumatic stress disorder) if it is created in cardiovascular system. The hydraulic energy redirection and release technology can successfully mitigate blast shock waves from the outer surface of the body armor. It should be further explored as an innovative approach to effectively protect against blast threats to civilian and military personnel.

## Introduction

In recent years, the usage of extremely powerful explosive devices in military operations and in terrorist attacks on civilian targets has become the hallmark of modern warfare and an increasing threat to both civilian and military personnel [1]. Blast injuries, as the result of physical trauma sustained in combat or terrorist explosions, are increasingly recognized and encountered worldwide. Explosive blast creates a defining supersonic over-pressurization shock wave [2]. A blast shock wave is a high-pressure area that expands rapidly outward from an explosive center as a sphere of compressed gases. It is characterized with a leading shock front of increased positive air pressure (which mainly causes damage at a distance from the explosion center) and a blast wind of negative air pressure (which follows immediately after the positive air pressure and sucks items back in towards the explosion center). Blast shock wave carries energy (an overpressure of 60–80 PSI) and can propagate at very high speeds (approximately 3,000–8,000 m/sec). The energy of a blast shock wave dissipates relatively quickly with distance [3]. When a blast shock wave interacts with a medium (solid, liquid, gas or plasma), the energy can be absorbed or transformed to kinetic energy of the medium that accelerates a body of a given mass from rest to its stated velocity. It initiates a retardation and energy absorbing process that captures the blast shock wave, and results in the rapid physical movement, displacement, deformation or breakage of the medium [4].

Personal protection against blast shock wave is currently the most difficult challenges facing body armor researchers and engineers. The rapid impact (compression) effects of blast shock wave on the human body may be the reason why fielded body armor and helmets can successfully prevent penetrating ballistic and stab injuries, but fail to mitigate against primary blast injuries caused by a blast shock wave. When blast shock wave acts on a soldier wearing currently standard fielded body armor, the personal armor is forced by surrounding overpressure to move rapidly toward the human body. The rapid impact of armors on the body, results in the non-penetrating injury, “Behind Armour Blunt Trauma” (BABT) [5]. Therefore, currently fielded body armor may not be able to protect the body against the impact of blast shock wave. In contrast, it can be possibly involved in coordination efforts with blast shock wave to increase the impact force to the body, causing more serious bodily injuries. Clearly, what is required to prevent and mitigate the primary blast injuries is an innovative body armor, which can protect effectively against the impact of blast shock wave.

In this study, the hydraulic energy redirection and release technology that aims to mitigate the impact of blast shock wave on the protected objects was developed based on the propagation and energy transfer characteristics of blast shock wave in a liquid [6]. Hydraulic energy is the power created by the compressive force or movement of a liquid in a confined area or a piping system. The power can be transferred and distributed through small tubes and flexible hoses, and be appropriately used for different machinery and tools such as hydraulic presses, hydraulic metal cutting machines, hydraulic excavators, and hydraulic forklift trucks. The hydraulic energy redirection and release technology employs the liquid-filled plastic tubings (which are similar to blood vessels filled with blood) as a blast pressure transformer to transfer kinetic energy of blast shock waves into hydraulic energy and to redirect and distribute hydraulic energy to the lower end of the liquid-filled piping system. This may cause blast overpressure to be released from the outer surface of the body armor or blast shelter through the openings at the lower end of the liquid-filled piping system without suffering damage to the human body. To evaluate the effectiveness of the hydraulic energy redirection and release technology on blast shock wave mitigation, the samples of the specifically designed blast-resistant body armor that were installed with the water-filled plastic tubings were tested.

## Methods

### Body Armor Test Sample

**Figure 1:**
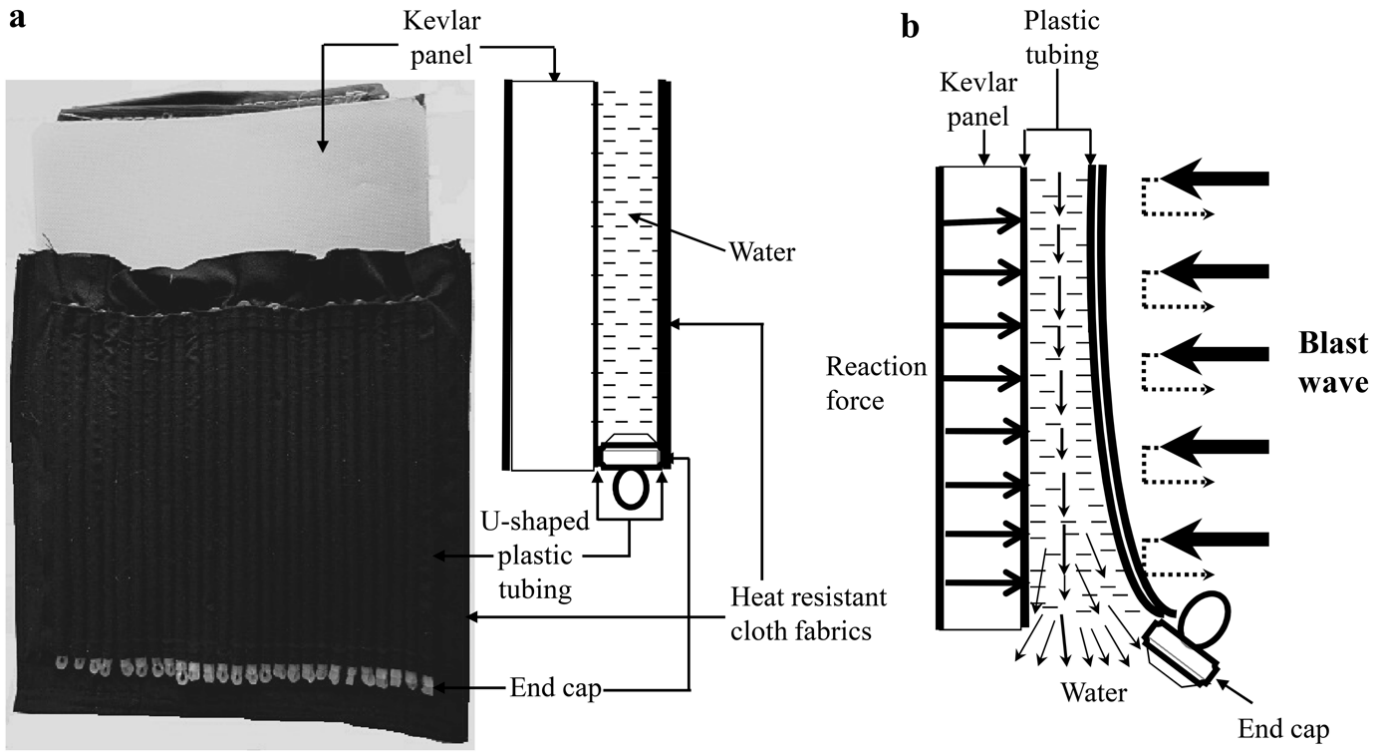
The structure of specifically designed body armor sample and its potential mechanism for blast shock wave mitigation. (a) A photograph showing the sample of the body armor that includes the water-filled plastic tubings, end caps, Kevlar panel, and heat resistant cloth fabrics; (b) A schematic diagram illustrating a side view of the sample of the body armor after a blast shock wave impacts the sample. The rapid compression effects of blast shock wave on the sample create an action force to compress water-filled plastic tubing and make it move against the Kevlar panel. Because the Kevlar panel is a hard plate fixed on a test frame, it will exert a reaction force against the plastic tubing. The action and reaction forces push water moving towards the lower end of the plastic tubing. Since water is incompressible, increased liquid pressure on the lower end forces the end cap to open and make water to spray out through the opening from the tubin, thus rapidly decreasing the liquid pressure inside of the tube.

The sample (a total weight of 0.505 kg, 20.3×20.3 cm^2^) of the specifically designed blast-resistant body armor was comprised of a layer of plastic tubings (Aqua Culture standard airline tubing, Wal-Mart Stores, Inc. Bentonville, Arkansas, USA) filled with water (a total weight of 0.23 kg), a dual purpose NIJ level 3A Kevlar panel (0.275 kg, 28 layers of DuPont Kevlar) that is currently used for ballistic (9 mm FMJ at 427 m/s minimum or 0.44 Magnum SJHP at 427 m/s minimum) and stab (25 joules minimum energy) protection (Meggitt Safety Systems, Inc., Simi Valley, California, USA), and the outer and inner layers of heat resistant clothing material (James Thompson & Co. Inc. New York, NY, USA) (Fig. 1a). The U-shaped water-filled plastic tubings were installed vertically in the body armor sample. The diameter and the length of a single plastic tubing were 4.8 mm and 203 mm, separately. The water volume in the single plastic tubing was 3.67 ml (the cross section area × the length). A tethered rubber cap (the end cap) was used to cover the opening at the lower end of each plastic tubing. The water-filled plastic tubings were employed as a blast pressure transformer to transfer, redirect and release blast kinetic energy from the outer surface of the sample. Kinetic energy of blast shock wave will be transferred into hydraulic energy in plastic tubings and then be quickly released with water flowing out of plastic tubings.

The rapid compression (squeezing) effects of blast shock wave on the sample of the specifically designed body armor may create an action force to compress the U-shaped plastic tubing installed in the sample and make it move against the Kevlar panel. Because the Kevlar panel is a solid hard plate fixed on the test frame, it will exert a reaction force against the plastic tubing. The action and reaction forces will push water inside the plastic tubing moving toward the opening at the lower end of the plastic tubing. Since water is incompressible, increased liquid pressure on the lower end of the tubing will knock off the end cap and force water to spray out through the opening from the tubing (Fig. 1b), thus quickly decreasing liquid pressure inside the plastic tubing.

### Experimental Setup and Pressure Measurement

**Figure 2:**
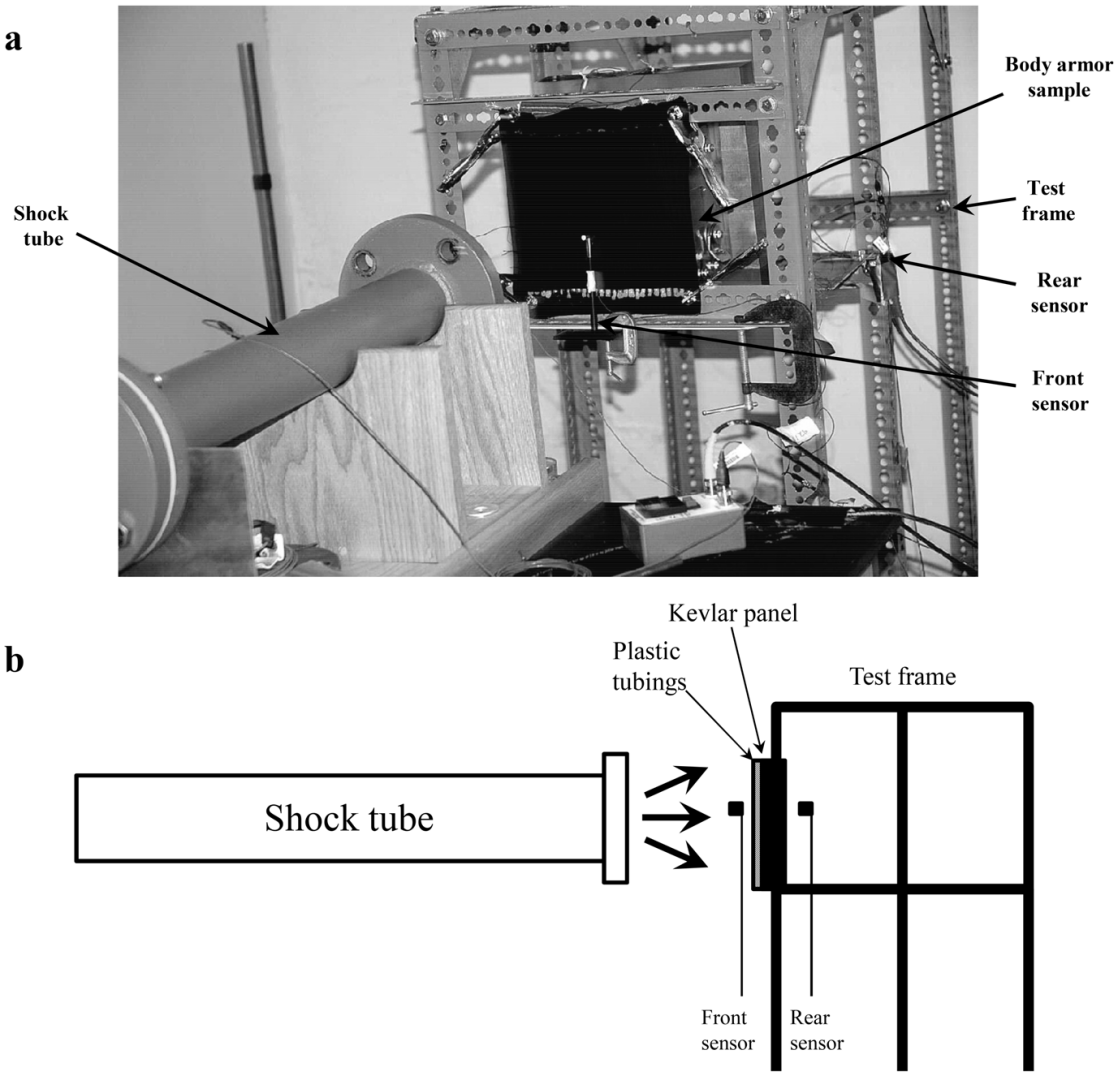
Experimental setup and the blast-testing equipments. (a) A photograph of the blast-testing equipment including shock tube, test frame, and front and sensors for assessment of the protective effects of the specially designed blast-resistant body armor against blast overpressure waves; (b) Schematic diagram of the blast-testing equipments. The sample of the body armor is fixed on a test frame that was approximately 40 cm away from the shock tube opening. The front sensor is placed 2 cm before the sample and the rear sensor is placed 4 cm behind the sample.

The samples of the body armor were tested to assess the protection against blast shock wave using PCB 901A10 gas-driven shock tube (15.24 cm in diameter, PCB Piezotronics, Inc. Depew, New York, USA). The peak pressures measured by a PCB incident pressure sensor (PCB Piezotronics, Inc., Depew, New York, USA) in driven section of the shock tube were about 140 psi. The sample was fixed on a test frame that was approximately 40 cm away from the shock tube opening (Fig. 2a). Two PCB 138M184 pressure sensors (Nominal pressure range is up to 500 psi, and pressure sensitivity is 10 mV/psi; PCB Piezotronics, Inc., Depew, New York, USA) were used as the front sensor and the rear sensor, respectively. The front sensor was placed 2 cm before the sample and the rear sensor was placed 4 cm behind the sample (Fig. 2b). The sensors were connected to a PCB Signal Conditioner (PCB Piezotronics, Inc., Depew, New York, USA) operating at 90 kHz sampling rate. The real time signal was recorded and processed with PXI Data Acquisition Systems (National Instruments, Austin, Texas, USA) connected to the signal conditioner.

## Results

**Figure 3:**
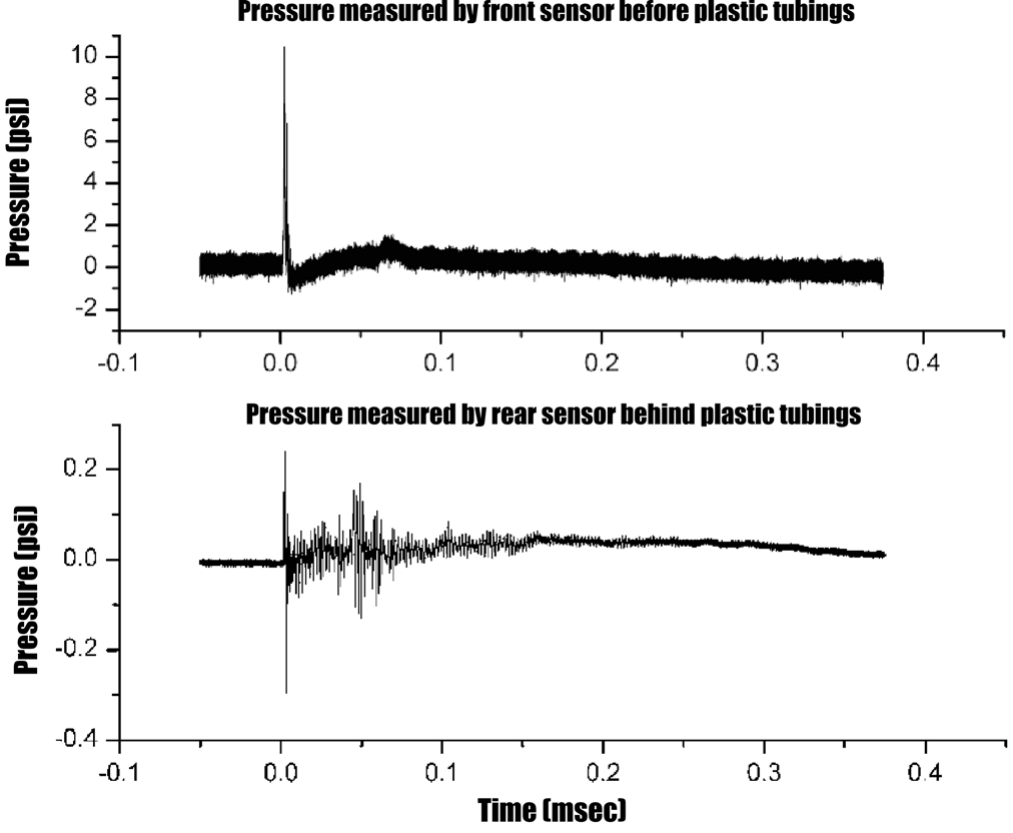
Actual pressure-time histories for both the front sensor and rear sensor during the first 0.4 msec after blast. The peak pressure of blast wave significantly decreases from 10.14 psi (pressure before plastic tubings) measured by the front sensor to 0.27 psi (pressure behind plastic tubings) measured by the rear sensor.

After a blast shock wave acted on the test sample, the action force of blast shock wave and the reaction force exerted by Kevlar panel compressed the water-filled plastic tubing and pushed water moving toward the opening at the lower end of the plastic tubing. Increased liquid pressure on the lower end of the tubing knocked immediately off the end cap and forced water to spray out through the opening, which resulted in a significant decrease in the peak pressure behind the plastic tubing layer alone by 97% (decreased from 10.14 psi measured by the front sensor to 0.27 psi measured by the rear sensor) in 0.2 msec (Fig. 3). After blast loading, about 0.22–0.37 ml (an average of 0.29 ml) of water were removed from a single plastic tubing through the opening, which meant that 6∼10% of total volume of water in the plastic tubings could be discharged in response to the impact of blast shock wave. This suggests that the discharge of 6∼10% of total volume of water from the plastic tubings may result in a 97% decrease in the peak pressure behind the test sample.

**Figure 4:**
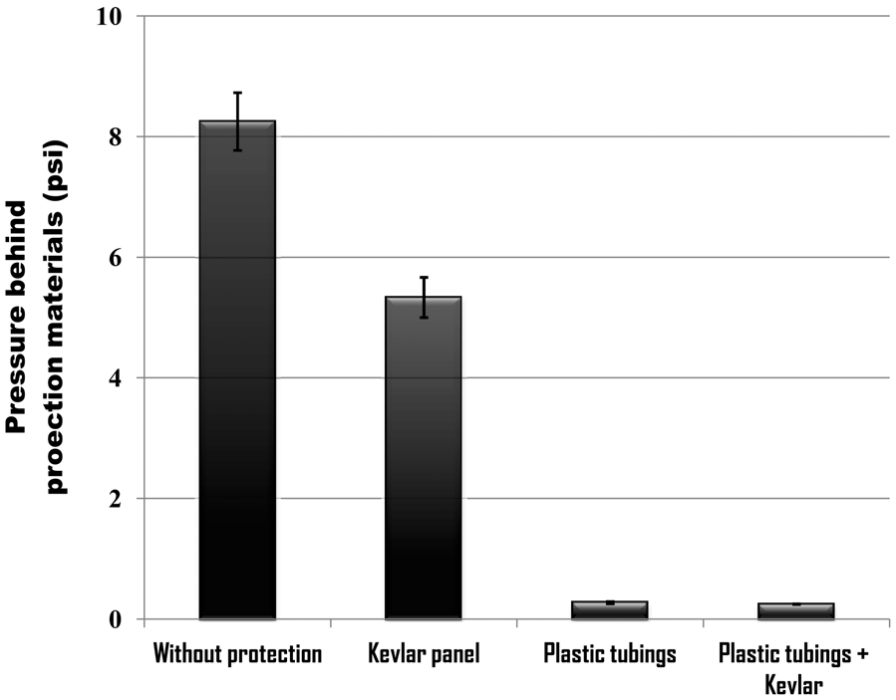
The peak pressures without protection material and behind different types of protection materials, measured using the rear sensor. The peak pressure measured without protection material was 8.25±0.48 psi (n = 4). The peak pressures behind Kevlar panel, plastic tubing layer and a combination of plastic tubing layer and Kevlar panel were 5.33±0.33 (n = 3), 0.27±0.02 (n = 5) and 0.25±0.004 psi (n = 4), respectively.

The peak pressures behind the test samples that were installed respectively with different protection materials were measured using the rear sensor. The peak pressure measured without the test sample was 8.25±0.48 psi. The peak pressures behind Kevlar panel, plastic tubing layer and a combination of plastic tubing layer and Kevlar panel were 5.33±0.33, 0.27±0.02 and 0.25±0.004 psi, respectively (Fig. 4). Kevlar panel alone is not effective against the blast shock wave itself. However, plastic tubing layer alone or a combination of plastic tubing layer and Kevlar panel can significantly mitigate the effects of blast shock wave on the test samples.

The results demonstrate that a major part of kinetic energy of the blast shock wave can be transferred into hydraulic energy in the water-filled plastic tubings that were installed vertically in the outer layer of the body armor sample. The hydraulic energy can be transmitted and distributed through the plastic tubings, and then be quickly released with water flowing out through the openings at the lower end of the plastic tubings. The blast mitigation technology, which employs a liquid-filled piping system to transfer, redirect and release kinetic energy of blast shock wave from the outer surface of the body armor or blast shelter, can successfully offer protection against blast shock waves and overpressure. The results also suggest that a volumetric water surge may be created when kinetic energy of blast shock wave is transferred into hydraulic energy in the water-filled plastic tubings to cause a rapid physical movement or displacement of the liquid. Based on the similar physical principle, a volumetric blood surge can possibly be created in human cardiovascular system if kinetic energy of blast shock wave is transferred into hydraulic energy in the closed blood piping system to cause a rapid movement or displacement of blood.

## Discussion

### 1. General Considerations for the Experimental Set-up

The compressed gas-driven shock tubes were generally used for blast injury research in rodent models and for preliminary, nondestructive testing of body armor materials [7], [8], [9]. The compressed gas-driven shock tube can imitate actual explosions and generate realistic blast shock waves in small-scale laboratory settings. It is easily operated and maintained in laboratory conditions. In this study, blast shock waves were generated using a compressed gas-driven shock tube to conduct blast tests for the samples of the specifically designed body armor. The compressed gas-driven shock tube was constructed of metal, in which a low-pressure gas in the driven section and a high-pressure gas in the driver section were separated using a 5-mil thick Mylar membrane. A pressure difference across the Mylar membrane caused the membrane to suddenly rupture, producing a blast shock wave propagating through the driven section to the test samples outside the shock tube opening. The pressure sensor mounted in the driven section (at the end of the shock tube) was used to verify the pressure that might be applied to the test sample for each exposure. The peak pressures of blast shock waves measured by the pressure sensor in driven section were about 140 psi. It was too high to apply for nondestructive testing of body armor samples if the test samples were fixed inside the shock tube.

Unlike previous wars, pulmonary and gastrointestinal barotraumas cases have been seldomly reported among soldiers deployed to Iraq and Afghanistan. Contrastly, traumatic brain injury (TBI) and post-traumatic stress disorder (PTSD) that can probably be induced by the lower blast pressures (less than the threshold pressure for barotraumas) have been considered as the signature injury of the wars in Iraq and Afghanistan. The threshold for pulmonary and gastrointestinal barotraumas is 13–15 psi of peak pressure [10], [11]. Hence, a peak pressure of 10 psi appears to be high enough to induce both TBI and PTSD in animal models and to conduct nondestructive testing of body armor materials. Because the peak pressures of blast shock waves generated by the shock tube could be controlled on a smaller scale by selection of the distance between the shock tube opening and the test sample [12], a distance of 40 cm away from the shock tube opening was set to get a peak pressure of about 10 psi applied to the samples of the specifically designed body armor. This is the reason why the test samples were fixed on a test frame that was 40 cm away from the shock tube opening in this study.

Because a blast shock wave propagates as a sphere of compressed gases at very high speed and has a leading shock front in the front surface of the sphere, the peak pressure measured by the front sensor that is placed the center of the sample represents the actual maximum pressure value of the blast shock wave interacting with the test sample. The difference between the peak pressures measured respectively by the front sensor and the rear sensor represents the kinetic energy loss of the blast shock wave in the test sample. Because there should be no significant difference among the peak pressures measured in a very short period of time (1–2 milliseconds) by different pressure sensors placed over a small-size test object, it is not necessary to place many sensors around the test object to measure the average diffusion rate of the shock wave distributed to all parts of the object or to obtain an average pressure curve over the entire object. Therefore, in both animal blast injury research and blast testing of body armor materials using the compressed gas-driven shock tubes, only one pressure sensor is generally placed the center of the test objects instead of many sensors placed all around the objects.

### 2. Hydraulic Energy Loss from the Plastic Tubings

In this study, the peak pressures behind the test samples have been found to reduce remarkably by 97% in 0.2 msec after discharged 6–10% (an average of 8%) of total volume of water from the plastic tubings. This suggests that it actually discharges only a small volume of the liquid from the plastic tubings to have significant hydraulic energy release from the outer surface of the body armor. A similar result has been reported by Stuhmiller [13]. In a simulation study using finite element modeling, a 294 psi blast loading against the torso produced 10 ml of blood flowing from the abdomen to the heart and 2 ml of blood flowing from the abdomen and the chest to the brain. Such a small volume of blood delivered to the brain could increase significantly intracranial pressure to nearly 147 psi. The results demonstrated that under blast loading, the hydraulic energy balance would be effectively and quickly achieved in the closed blood piping system if a small volume (2–10 ml) of blood could be delivered to distant organs or tissues.

The reasons why the discharge of a small volume of the liquid from a piping system can result in a significant decrease in hydraulic energy level in the system is not entirely clear. When a blast load of 10 psi acts on the plastic tubings, its energy may be absorbed and transformed to kinetic energy of water, which will accelerate the water from rest to a high velocity in a very short time (within 0.2 msec). If the distance of the water flow in the single plastic tubing is 16 mm ( =  the average amount of water discharged from the tubing ÷ the cross section area × 1000  =  0.29 ml/18.09 mm^2^ × 1000) in 0.2 msec after blast loading, the water flow velocity (V) will be 80 m/sec. The sudden increase in water flow velocity may carry and deliver most of hydraulic energy toward the opening at the end of the plastic tubing. Because the cross section area of a single plastic tubing is 18.09 mm^2^ (the inside tubing diameter is 4.8 mm), water flow rate (Q) will be 0.52 m^3^/h (28800 m/h × 0.00001809 m^2^). According to Darcy-Weisbach equation [14], the energy loss (or head loss) gradient due to a water flow (friction) in a plastic tubing less than 125 mm in diameter can be evaluated as follows:
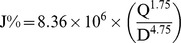



J  =  energy loss (%)

Q  =  water flow rate (m3/hour)  =  cross section area of a tubing (A) times water flow velocity (V)

D  =  inside tubing diameter (mm)

The energy loss gradient at the end of the plastic tubing is as follows:
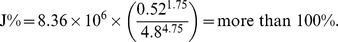



It means almost all hydraulic energy can possibly be lost after water flowing out of its appropriate volume through the opening. This may be a possible mechanism by which the hydraulic energy balance can be achieved by discharging a small volume of the water from the plastic tubings following blast loading.

### 3. Transferring Kinetic Energy of the Blast Shock Wave into Hydraulic Energy in the Water to Cause the Volumetric Water Surges

A liquid (such as water, blood, alcohol, juice, milk, oil, etc.) is essentially incompressible and does not absorb energy from other sources to an appreciable extent. At the same time, the liquid is an important transmission medium that is capable of moving high-pressure loads and transferring kinetic energy to other objects due to its incompressibility [15]. A tsunami generated by an earthquake, a volcanic eruption, or an underwater nuclear blast is a typical example of transferring kinetic energy of natural or man-made disaster into hydraulic energy to cause a rapid physical movement or displacement of a large volume of a body of water in an ocean or a large lake [16], [17]. When these disasters occur beneath the sea, the shock waves produced by the disasters radiate out and cause the water above the disaster area to be displaced from its equilibrium position, which forms tsunami waves (or called huge volumetric water surges) [18]. Tsunami waves travelling at high speed have an enormous destructive power. When a tsunami wave approaches a shoreline, it can drain off buildings and objects in coastal areas and carry all with it, even if the wave does not look large.

Two cases of underwater explosions also demonstrated that kinetic energy of blasts can be transferred into hydraulic energy in water to cause a noncontact damage to the ship’s hull and human skull. A jet of water (a volumetric water surge) caused by an underwater torpedo explosion resulted in the ROKS Cheonan some distance away from the explosion site to break and sank off the coast South Korea on March 26, 2010 [19]. The underwater torpedo explosion creates a rapidly expanding gas bubble in the water, and this bubble will collapse from the bottom owing to the difference in pressure. This creates a jet of water that shoots out in all directions and can go sideways over a hundred meters to strike a ship and to cause a noncontact damage to the ship’s hull [20]. Another case is that, a firecracker (which was approximately equal to three grams of TNT in explosive power) generated a very strong jet of water with impact energies between 440 to 1800 in.-lb and impact impulses between 1.8 to 3.5 lb/sec, to cause extensive comminuted skull fractures, severe brain injury, and death after it exploded in close proximity to the victim’s head in water [21]. Without water acting as a pressure transmission medium to transfer kinetic energy of blasts to other objects, a blast shock wave that is generated by explosion itself is not able to cause a remote damage to the ship’s hull and human skull from a relatively long distance.

**Figure 5:**
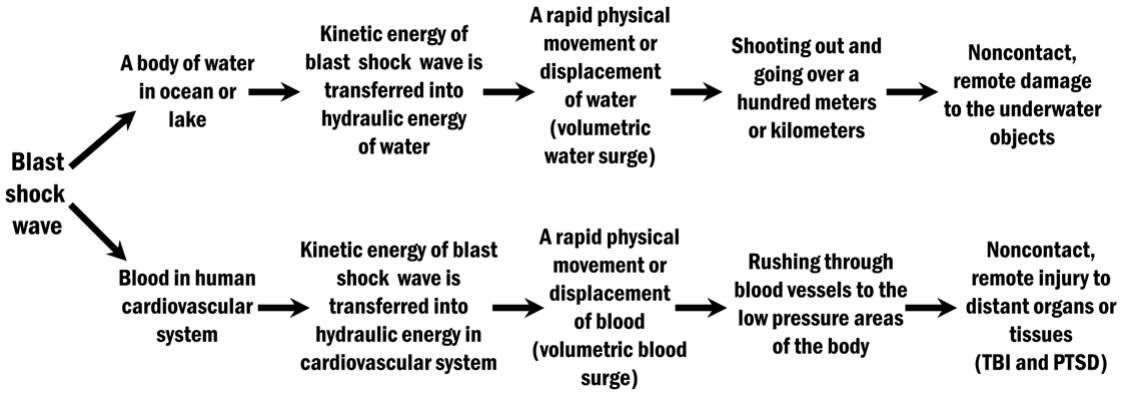
The potential mechanism by which the blast shock wave causes noncontact, remote damage to the underwater objects (such as ships, underwater animals and humans) through water, and to the human organs or tissues through blood.

The volumetric water surge, which is represented as a form of hydraulic energy transmissions in water, can generate an astounding destructive power to any objects that may be encountered in its motion path (Fig. 5). This is the reason why many ships (naval surface combatants, subs and commercial tankers) were totally disabled by noncontact underwater explosions during both World War II and the period after World War II [22], and also the reason why blast shock wave generated by an underwater explosion could cause serious noncontact, remote damage or death to underwater animals and humans swimming in water.

### 4. Transferring Kinetic Energy of the Blast Shock Wave into Hydraulic Energy in Human Cardiovascular System to Cause a Volumetric Blood Surge

When a blast shock wave acts on the human body, the surrounding overpressure causes a sudden, transient (1–2 milliseconds) increase in atmospheric pressure on the organs and the cardiovascular system. Elevated overall pressure on the cardiovascular system will rapidly raise blood pressure to cause a rapid physical movement or displacement of blood (a volumetric blood surge) [23]. This will result in kinetic energy of blast shock wave to be transferred into hydraulic energy in the cardiovascular system. The hydraulic energy will be further transmitted through blood vessels to the low-pressure areas of the body where the atmospheric pressure is below the areas that are predominantly affected by blast shock wave. As a form of hydraulic energy transmissions, the volumetric blood surge in human cardiovascular system has a strong destructive power to human organs and tissues. It can rush through blood vessels to the low pressure areas of the body, causing a noncontact, remote injury to distant organs or tissues (Fig. 5).

The volumetric blood surge should be the major contributor to non-impact blast-induced TBI and PTSD. Because human skull can resist compression of a blast shock wave to avoid a sudden increase in intracranial pressure, the cranial cavity should be a relatively low pressure area when a blast shock wave acts on the whole human body. Therefore, the volumetric blood surge will certainly move through blood vessels to the low-pressure cranial cavity from the high-pressure body cavity. It will dramatically increase cerebral perfusion pressure and cause damage to both tiny cerebral blood vessels and the BBB. Cerebrovascular insults and the BBB damage caused by the volumetric blood surge will trigger secondary neuronal damage [23]. Secondary neuronal damage is an indirect consequence of initial injury and a major contributor to the ultimate neuronal cell death and neural loss in the injured brain. This delayed secondary neuronal damage has been considered to be largely responsible for serious neurological and psychiatric impairments, including memory loss, inability to concentrate, speech problems, motor and sensory deficits, and behavioral problems [24].

### 5. Protection Against Blast Shock Waves Using the Hydraulic Energy Redirection and Release Technology

Several experimental studies have shown that the effects of blast shock wave can be mitigated by water mists (or walls) [25], [26], [27], [28]. However, the blast mitigation using water mists may be used only for blast shelter, not for blast-resistant personal armors. When a blast shock wave acts on a liquid-filled piping system, a sudden increase in pressure on the wall of the system will increase dramatically liquid pressure inside the system to cause a rapid physical movement or displacement of the liquid (a volumetric liquid surge). This will transfer kinetic energy of blast shock wave into hydraulic energy in the system. The hydraulic energy can be further redirected and distributed through the piping system to a desired target area, and then be quickly released if the liquid is able to flow partially out of the system through the openings.

In our preliminary blast tests, the samples of specifically designed body armor were fixed on the test frame to evaluate the abilities of the liquid-filled plastic tubings (which look like a layer of artificial blood vessels around the human body) to migrate the impact of blast shock wave on the samples. The results demonstrated that the liquid-filled plastic tubings could effectively absorb, redirect and release kinetic energy of the blast shock wave before the Kevlar panel, thus significantly reducing the rapid compression effects of blast shock wave on the samples. The unique blast protective characteristics of the liquid-filled piping system enable to further develop the hydraulic energy redirection and release technology for mitigating the effects of blast wave on the human body. The new-generation body armor, helmets, combat boots, and other gear that are developed using the hydraulic energy redirection and release technology will combine blast and ballistic protection capabilities to achieve superior performance against both blast and ballistic threats. The new-generation personal armor may include an outer layer of heat-resistant clothing material, a layer of liquid-filled plastic tubings that are inserted into semi-circular troughs or grooves in a lightweight metal alloy plate, a layer of packing foam, a Kevlar panel, springs that pass through the packing foam layer and are mounted between the metal alloy plate and Kevlar panel, and an inner layer of common clothing material. Such an innovative overall design will guarantee the liquid flows effectively out of the plastic tubings through the openings while the plastic tubings interact with both the impact (action) force which is created by blast shock wave and the reaction force which is exerted by hard plates (lightweight metal alloy plate and Kevlar panel) and is then increased by springs. It will reach the same blast migration effect as the samples fixed on the test frame. This design will allow the new personal armor to have a relatively low weight, to be easily worn and carried, and to be used for extreme temperature environments. In addition, the specifically designed body armor will not significantly influence the mobility and movement of the human body, and allows war fighters to maintain mission capability.

The hydraulic energy redirection and release technology incorporates a particularly innovative feature to block the direct transfer of kinetic energy of blast shock wave to cardiovascular system to cause a volumetric blood surge, thus protecting against noncontact, remote injuries caused by a blast shock wave, such as non-impact, blast-induced TBI and PTSD. The technology has the potential usefulness for not only developing next-generation body armor, helmets, the extremity body armor, but also blast protection of the warship’s hulls, military armored vehicles and bomb shelters.
