# Correction: Blast Shock Wave Mitigation Using the Hydraulic Energy Redirection and Release Technology

**DOI:** 10.1371/annotation/f1eb267c-5d8d-4e32-af75-462f20353732

**Published:** 2012-09-05

**Authors:** Yun Chen, Wei Huang, Shlomi Constantini

There is an error in the competing interest statement. The correct statement is: The hydraulic energy redirection and release technology described in this manuscript was filed with US PTO for a patent application, and the US PTO has disclosed the invention to the public on June 14, 2012 (Publication No. 2012/0144988). This does not alter the authors' adherence to all the PLOS ONE policies on sharing data and materials. 

